# The Journey Toward Establishing Inpatient Care for Small and Sick Newborns in Ethiopia, India, Malawi, and Rwanda

**DOI:** 10.9745/GHSP-D-22-00510

**Published:** 2023-08-28

**Authors:** Patricia S. Coffey, Kiersten Israel-Ballard, Laura Meyer, Kimberly Mansen, Nesibu Agonafir, Mitiku Bekere, Queen Dube, Gerard Kaberuka, Jayendra Kasar, Aishwarya Kharade, Sudhir Maknikar, Kelsang Choeki Namgyal, Alinane Linda Nyondo-Mipando, Stephen Rulisa, Bogale Worku, Cyril Engmann

**Affiliations:** a PATH, Seattle, WA, USA.; b PATH, Addis Ababa, Ethiopia.; c Consultant, Addis Ababa, Ethiopia.; d Ministry of Health, Lilongwe, Malawi.; e Consultant, Kigali, Rwanda.; f PATH, Delhi, India.; g Consultant, Delhi, India.; h Kamuzu University of Health Sciences, Blantyre, Malawi.; i University of Rwanda, Kigali, Rwanda.; j Ethiopian Pediatric Society, Addis Ababa, Ethiopia.; k University of Washington, Seattle, WA.; l Seattle Children’s Hospital, Seattle, WA.

## Abstract

**Background:**

Limited information is available about the approaches used and lessons learned from low- and middle-income countries that have implemented inpatient services for small and sick newborns. We developed descriptive case studies to compare the journeys to establish inpatient newborn care across Ethiopia, India, Malawi, and Rwanda.

**Methods:**

A total of 57 interviews with stakeholders in Ethiopia (n=12), India (n=12), Malawi (n=16), and Rwanda (n=17) informed the case studies. Our heuristic data analysis followed a deductive organizing framework approach. We informed our data analysis via targeted literature searches to uncover details related to key events. We used the NEST360 Theory of Change for facility-based care, which reflects the World Health Organization (WHO) Health Systems Framework as a starting point and added, as necessary, in an edit processing format until data saturation was achieved.

**Findings:**

Results highlight the strategies and innovation used to establish small and sick newborn care by health system building block and by country. We conducted a gap analysis of implementation of WHO Standards for Improving Facility-Based Care. The journeys to establish inpatient newborn care across the 4 countries are similar in terms of trajectory yet unique in their implementation. Unifying themes include leadership and governance at national level to consolidate and coordinate action to improve newborn quality of care, investment to build staff skills on data collection and use, and institutionalization of regular neonatal data reviews to identify gaps and propose relevant strategies.

**Conclusion:**

Efforts to establish and scale inpatient care for small and sick newborns in Ethiopia, India, Malawi, and Rwanda over the last decade have led to remarkable success. These country examples can inspire more nascent initiatives that other low- and middle-income countries may undertake. Documentation should give voice to lived country experience, not all of which is fully captured in existing, peer-reviewed published literature.

## Abstract (precis)

Documenting the journey to establish inpatient care for small and sick newborns in Ethiopia, India, Malawi, and Rwanda, the authors showcase the remarkable progress and share lessons with stakeholders in other countries who aim to do the same.

## Teaser

**Key Findings:**

- Each country’s unique journey to establish inpatient care and roll out service delivery for small and sick newborns shows the diversity of actions and actors required to scale a new practice across different geographies:
  - In Ethiopia, care was implemented in a stepwise expansion from a newborn care corner to community-based newborn care to neonatal intensive care units.
  - In India, a model newborn care unit was an exemplar for national scale.
  - In Malawi, a hub-and-spoke model used the introduction of continuous positive airway pressure as a grounding point.
  - In Rwanda, integrated small and sick newborn care was initiated at the district level in the eastern region of the country, far from the capital city.

**Key Implications:**

- National stakeholders should document country-level strategies and innovation related to the establishment of small and sick newborn care, thereby giving voice to lived country experience.
- Program managers can use the learnings from various countries to establish or strengthen small and sick newborn care service delivery.

## BACKGROUND

Over the past 3 decades, significant improvements in newborn care have occurred. Since 2000, global neonatal mortality has virtually halved, from 4 million newborn deaths annually to slightly more than 2 million deaths in 2021.^1^ These improvements have been achieved through a variety of factors: increased leadership, focus, and funding; strengthened programmatic realignments and integration of newborn health into traditional reproductive, maternal, and child health programs; concerted research and measurement initiatives; numerous technological innovations; and heightened evidence-based advocacy efforts. Both the Lancet Neonatal Survival^2^ and Every Newborn series^3^ provided the evidence base to raise awareness of the need to prioritize newborn mortality and morbidity. These heightened evidence-based advocacy efforts, particularly the Every Newborn Action Plan (ENAP),^4^ created common goals, targets, and metrics and, most importantly, fostered adoption and ownership of newborn health at national and subnational levels. In their entirety, these initiatives^5^^–^^7^ offered an innovative vision for inpatient newborn care. However, there is a paucity of in-depth practical guidance and information about the approaches used and lessons learned in low- and middle-income countries (LMICs) as they designed and implemented their inpatient newborn services for small and sick newborns.^8^

Several countries are making concerted efforts to strengthen inpatient newborn care units for small and sick newborns. Complementary efforts at the international level include the creation of guidance, such as the recently released World Health Organization (WHO) Standards for Improving the Quality of Care for Small and Sick Newborns in Health Facilities.^9^ At the country level, the descriptions of the approaches taken and evaluation of their success have not been comprehensively documented, analyzed, and communicated. This case study describes holistically and chronologically the journey undertaken in 4 countries (Ethiopia, India, Malawi, and Rwanda) to establish and scale inpatient newborn care uniquely from the country perspective.

Several countries are making concerted efforts to strengthen inpatient small and sick newborn care, but their approaches have not been comprehensively documented.

## METHODS

### Case Study Approach

We articulate a purely country-focused perspective to describe how inpatient care for small and sick newborns was established. This work does not attempt to assess how well small and sick newborn care services are now performing in these countries, but rather it showcases how these services came to be.

We developed descriptive, multiple case studies to identify and compare the journeys to establishing inpatient newborn care across these countries. The funding agency purposively selected these 4 geographies to capture countries with some advanced experiences in establishing and scaling services for small and sick newborns. Data collection included a scoping review of published and gray literature (including key newborn care guidelines and clinical protocols) to document national and subnational policies and scale-up strategies, in-person or virtual in-depth interviews with key newborn stakeholders using semistructured standardized questionnaires, follow-up email correspondence, and data validation webinars with national stakeholders in each country.

To start this case study process, we sought people who were the core drivers for establishing small and sick newborn care in each country, regardless of their institutional affiliation. Some of these individuals had been or were currently affiliated with the ministry of health at national or subnational levels at the time of the interview. Our main recruitment criterion was that the key informant had been integrally involved in the process of establishing small and sick newborn care over the last decade and, thereby, had been part of a nucleus of stakeholders in each country that drove implementation of small and sick newborn care programming.

Our sampling and recruitment methods included criterion sampling to identify published and gray literature for the scoping review, intensity sampling to identify an initial core set of key informants who could provide in-depth information about the establishment of small and sick newborn care in each country, and respondent-driven sampling to identify additional key stakeholders that could fill in specific gaps in the overall case study. We recruited key informants through personalized email and telephone contact. Stakeholders gave their verbal consent to be interviewed after they were informed about the purpose and description of the study.

**FIGURE 1:**
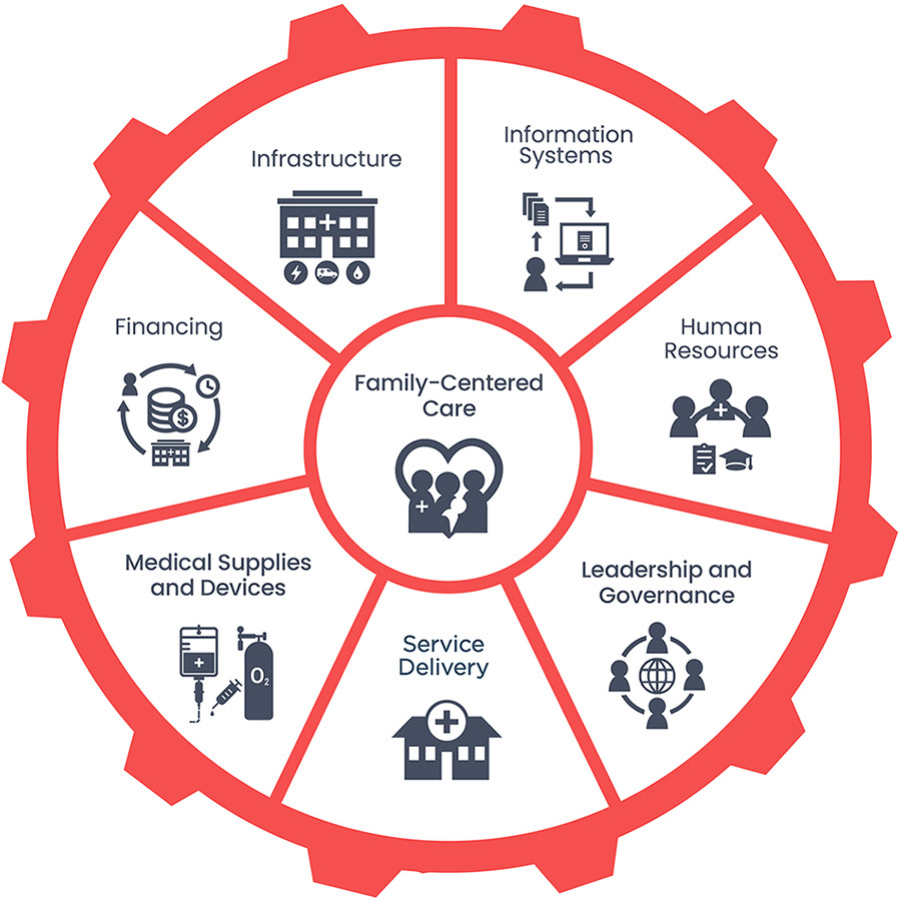
WHO Health System Building Blocks Used as Organizing Principle for Case Studies on Small and Sick Newborn Care in 4 Countries^12^ Abbreviation: WHO, World Health Organization. Credit: © NEST360/UNICEF Implementation Toolkit for Small and Sick Newborn Care.

Our data analysis was heuristic and followed a deductive organizing framework approach. We informed our data analysis via targeted literature searches to uncover details related to key events. We used the NEST360 Theory of Change for facility-based care, which reflects the WHO Health Systems Building Blocks Framework as a starting point and added, as necessary, in an edit processing format until data saturation was achieved. Saturation refers to the point during data analysis at which incoming data points (interviews) produce little or no new useful information relative to the objective of our case study. The WHO Health Systems Framework^10^ can identify specific entry points to improve the implementation of newborn interventions at critical health system building blocks: leadership and governance, financing, workforce, information systems, and essential medical commodities and was adapted by NEST360 to include family-centered care and by our team to include service delivery (Figure 1).^11^ This framework was also adapted by the ENAP to assess bottlenecks to implementing critical newborn care interventions and packages.^12^

### Ethical Approval

The Research Determination Committee at PATH reviewed this case study protocol and determined that it is not human subjects research.

## FINDINGS

A total of 57 interviews with key stakeholders in Ethiopia (n=12), India (n=12), Malawi (n=16), and Rwanda (n=17) informed the case studies. At the time of the interviews, stakeholders were working as health care providers or program planners from the ministry of health at various levels, nongovernmental organization implementing partners, professional associations, or private facilities. In this overview publication, we focus on strategies used, innovations implemented, and approaches used to roll out service delivery.

**FIGURE 2:**
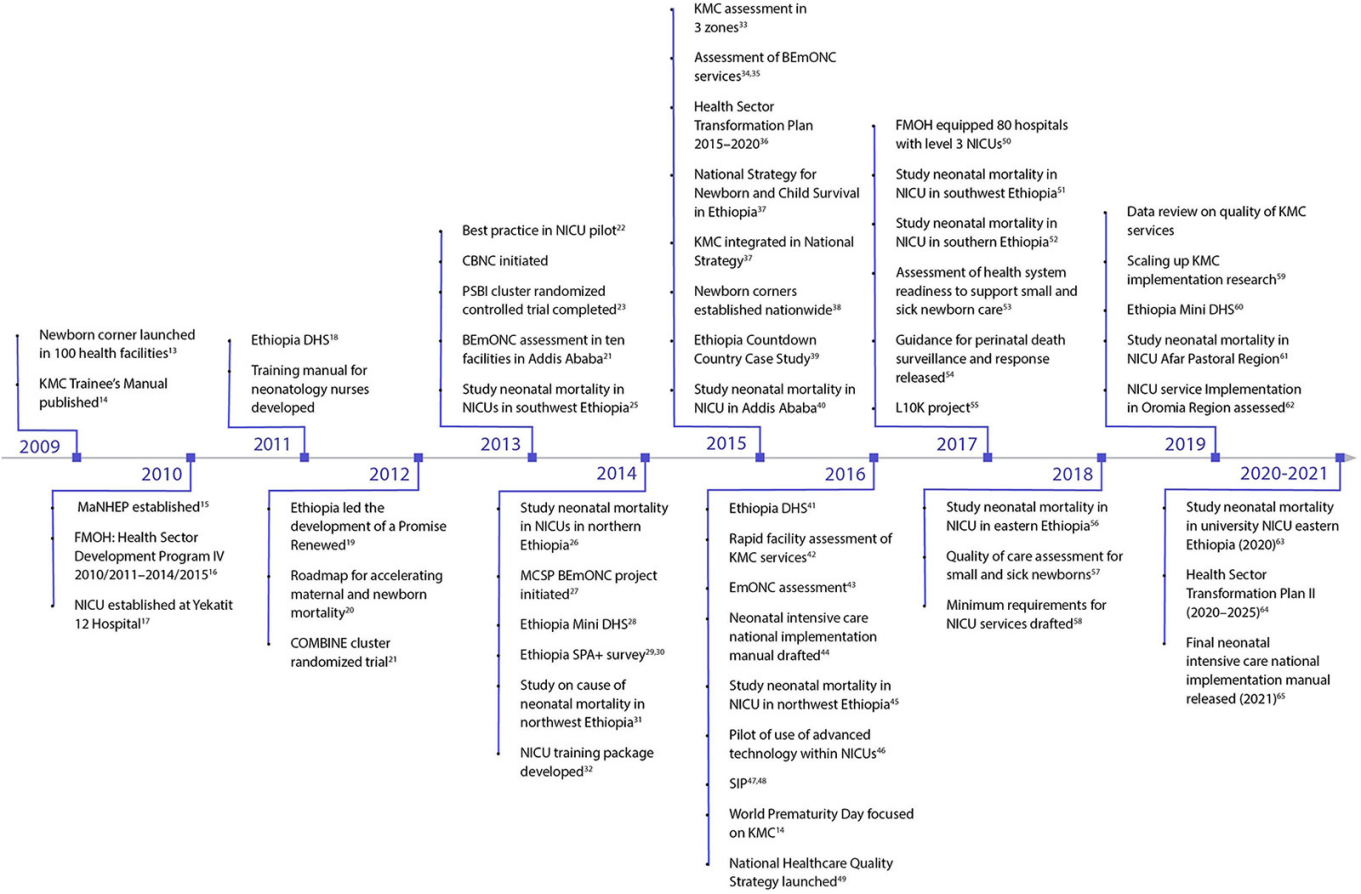
Ethiopia Timeline Showing Significant Activities Undertaken to Establish Small and Sick Newborn Care, 2009–2021 Abbreviations: BEmONC; basic emergency obstretric and newborn care; CBNC, community-based newborn care; DHS, Demographic and Health Survey; EmONC; emergency obstetric and newborn care; FMOH, Federal Ministry of Health; KMC, kangaroo mother care; L10K, Last 10 Kilometers; MaNHEP, Maternal and Newborn Health Ethiopia Partnership; MCSP, Maternal and Child Survival Program; NICU, newborn intensive care unit**;** PSBI, possible severe bacterial infections; SIP, Study of Illness in Preterms; SPA+, Service Provision Assessment Plus.

**FIGURE 3:**
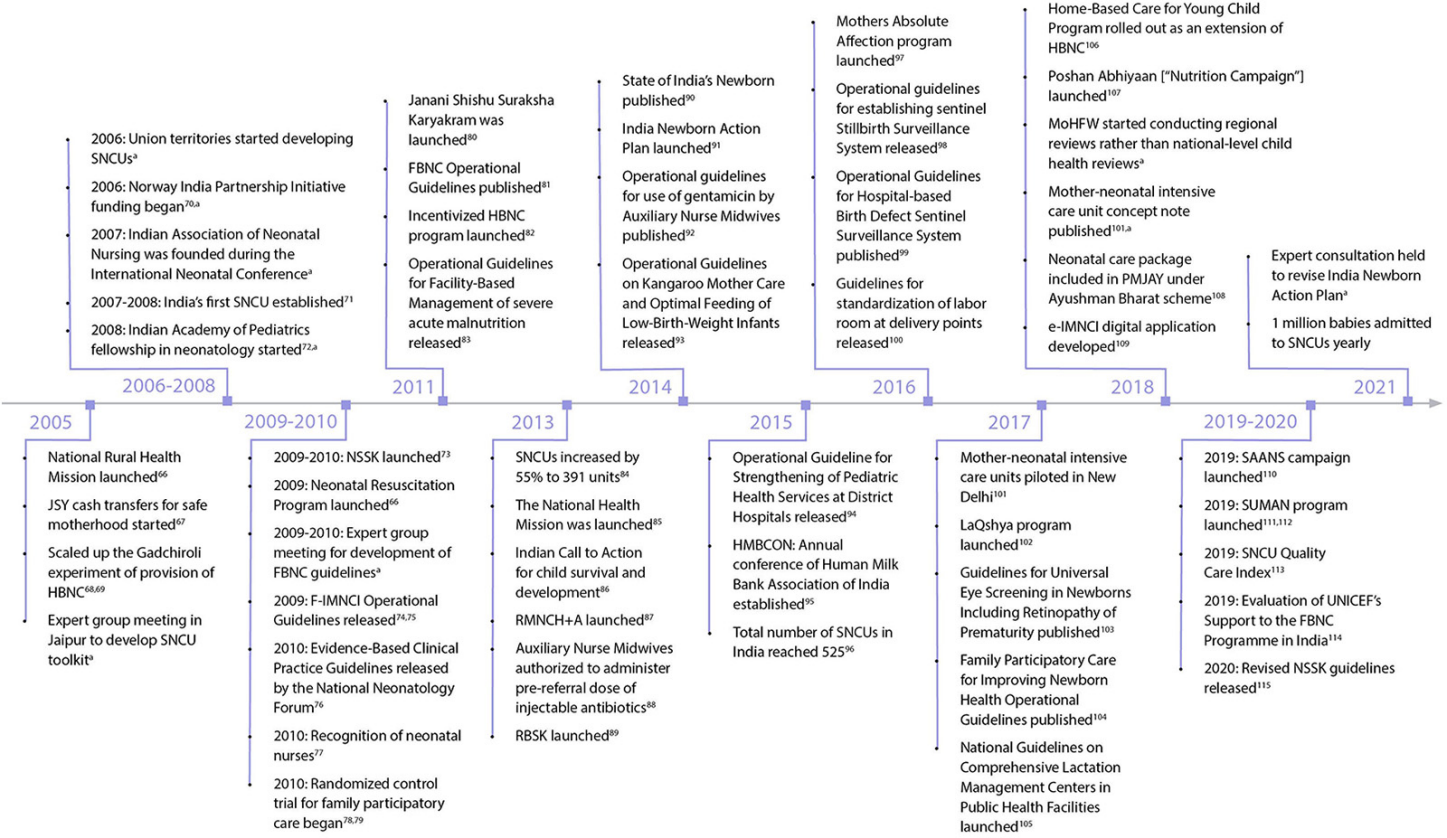
India Timeline Showing Significant Activities Undertaken to Establish Small and Sick Newborn Care, 2005–2021 Abbreviations: HBNC, home-based newborn care; HMBCON, Human Milk Bank Conference; F-IMNCI; Facility Based Integrated Management of Neonatal and Childhood Illness; JSY, Janani Suraksha Yojana; NSSK, Navajat Shishu Surkisha Karyakram; RBSK, Rashtriya Bal Swasthya Karyakam; RMNCH+, reproductive, maternal, newborn and child health plus; SAANS, Social Action and Awareness to Neutralize Pneumonia; SNCU, special newborn care unit.

**FIGURE 4:**
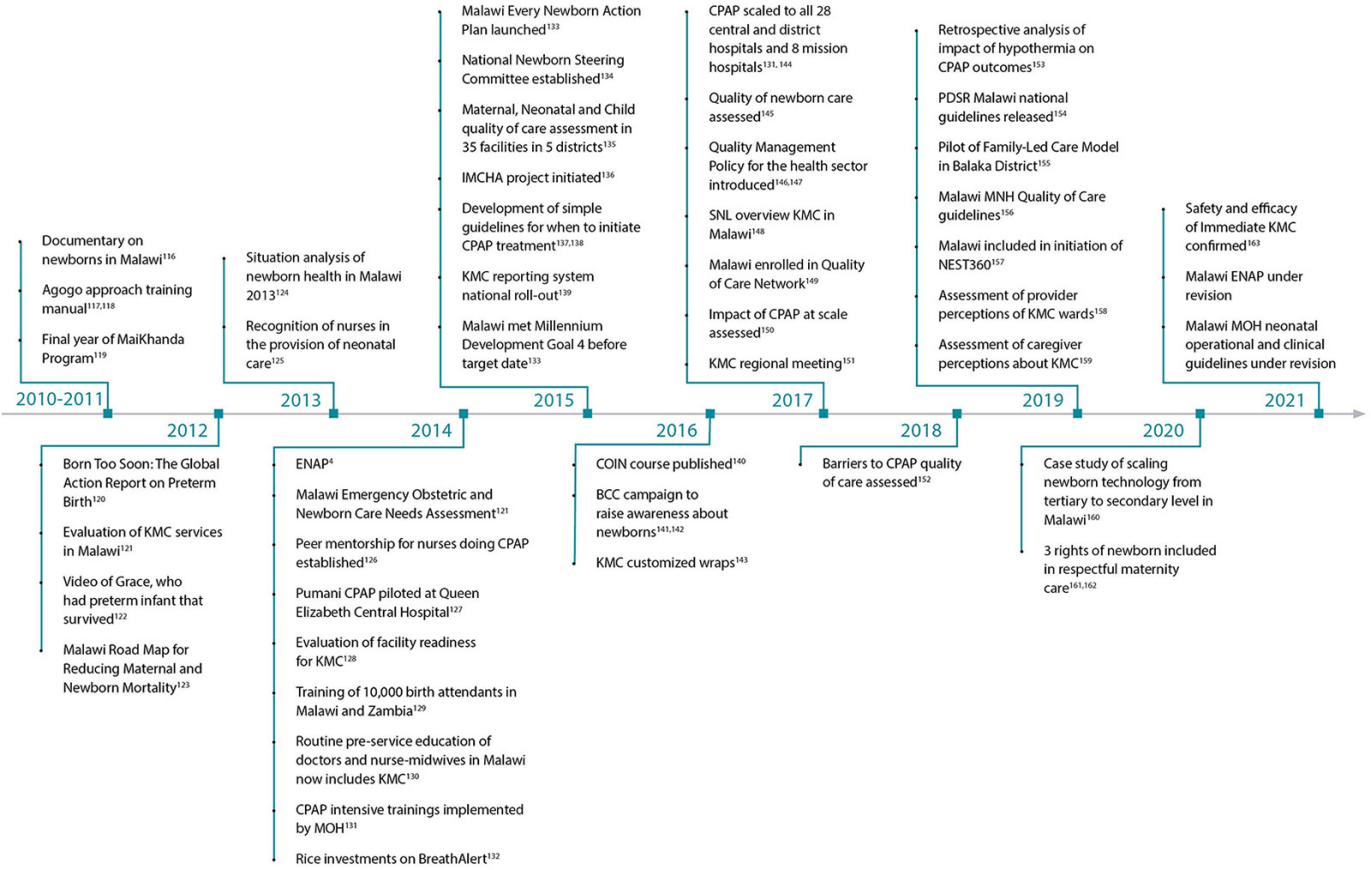
Malawi Timeline Showing Significant Activities Undertaken to Establish Small and Sick Newborn Care, 2010–2021 Abbreviations: BCC, behavior change communication; COIN, Care for the Infant and Newborn; CPAP, continuous positive air pressure; ENAP, Every Newborn Action Plan; IMCHA, Innovating for Maternal and Child Health; KMC, kangaroo mother care; MNH, maternal and newborn health; MOH, Ministry of Health; PDSR, perinatal death surveillance and response; SNL, Saving Newborn Lives.

**FIGURE 5:**
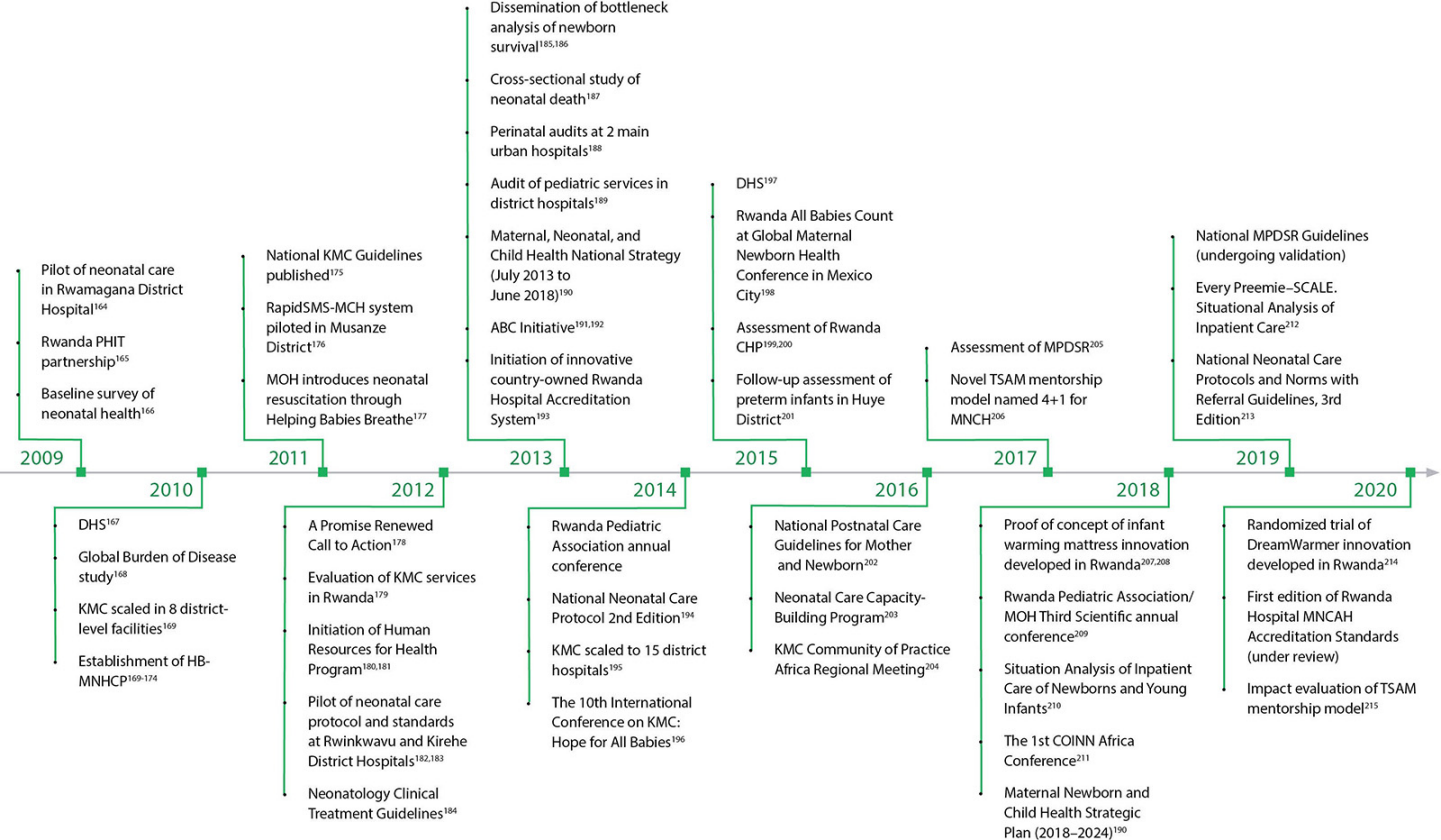
Rwanda Timeline Showing Significant Activities Undertaken to Establish Small and Sick Newborn Care, 2009–2020 Abbreviations: ABC, All Babies Count; CHP, community health program; COINN, Council of Internal Neonatal Nurses; DHS, Demographic and Health Survey; HB-MNCHP, home-based maternal and neonatal health care package; KMC, kangaroo mother care; MCH, maternal and child health; MPDSR, maternal and perinatal death surveillance and response; MNCAH, maternal, newborn, child, and adolescent health; MNCH, maternal, newborn, and child health; MOH, Ministry of Health; PHIT, Population Health Implementation and Training; TSAM, Training Support and Access Model.

We created an event timeline that shows the significant activities that were undertaken over the last decade to establish a system of care for small and sick newborns in Ethiopia (Figure 2),^13^^–^^65^ India (Figure 3),^66^^–^^115^ Malawi (Figure 4),^116^^–^^163^ and Rwanda (Figure 5).^164^^–^^215^ The timeline tracks each country’s efforts, which range from the inception of planning for care for small and sick newborns through activities to operationalize and scale inpatient care.

### Strategies Used to Establish and Scale Small and Sick Newborn Care

Strategies used to establish and scale small and sick newborn care varied by country (Table 1).^216^^–^^221^ Overall, the broad approaches used were fairly similar across countries, although each strategy has its unique permutations. Overall, good governance, commitment, and leadership at the national level helped drive improvements in care. Building the capacity of a specialized workforce, measuring neonatal-specific indicators, and adapting existing infrastructure were used as strategies to support small and sick newborn service delivery across all countries.

**TABLE 1.** Strategies to Establish Small and Sick Newborn Care, by Health System Building Block and Country

Health System Building Block	Ethiopia	India	Malawi	Rwanda
Leadership and governance	Improve ownership, leadership, and accountability through Ministry of Health-led partnerships and coordination platforms for newborn and child survival.^16^^,^^17^	Coordinate systematic implementation of small and sick newborn activities led by national (financial and technical guidance), state, and district health departments (planning and implementation) using national FBNC guidelines and training modules.^81^	Oversight of small and sick newborn care led by newborn focal point at the Ministry of Health.^4^^,^^134^	Government of Rwanda provides strong leadership and accountability.
Human resources	Accelerate health workforce development through in-service training, mentorship, and continuing professional development of mid-level health workforce.	Develop health workforce with the necessary skills to provide the appropriate level of care through specialized training courses for FBNC.^77^	Build capacity among available personnel to create a pool of specialized health workers to deliver inpatient care and are retained within the setting by embedding neonatal care in preservice training and specialized courses decisions to scale up care to prevent neonatal mortality.^140^	Build capacity of specialized workforce to deliver small and sick newborn care through a learning collaborative model where doctors, nurses, community health workers, and political leaders share data and take appropriate decisions to scale up care to prevent neonatal mortality.^165^^,^^180^^,^^181^
Information systems	Include neonatal care indicators in the HMIS to ensure data use for informed decision-making.	Monitor measurable indicators to inform health policy and programs on newborn health.	Capture all neonatal-related indicators within the health management information system.	Integrate data collection, monitoring, and use at all levels of care and building capacity of those using data to make life-changing decisions for improving the quality of small and sick newborn care services.
Infrastructure	Standardize and build appropriate infrastructure for NICU and newborn care at hospitals and health centers, respectively.	Establish nationwide network of NBCCs at every point of childbirth, NBSUs at first referral units, and SNCUs at district hospitals.^71^^,^^84^	Advocate that all hospitals to be built and those being renovated should have a purpose-built neonatal unit included in it.	Adapt existing infrastructure for small and sick newborn care according to available budgets.
Health system financing	Subsidize the cost of care in public health facilities and implement community-based health insurance schemes.^216^	Support states with financial resources in 60:40 (national/state) sharing ratio using annual program implementation plans to develop budgets for FBNC.	Ensure that funding for small and sick newborn care is allocated at the district and facility levels.	Fully fund small and sick newborn care services through universal health coverage.
Medical supplies and devices	Ensure availability of essential medical supplies and devices for NICU through an Integrated Pharmaceutical Logistic System.	Encourage collaboration of neonatologists, engineers, and entrepreneurs to produce and supply high-quality neonatal equipment of several high-volume categories at affordable cost.^217^^–^^221^	Maintain adequate inventory of equipment and supplies for management of small and sick newborns in the central medical stores.^121^^,^^128^	Provide centralized guidance around the small and sick newborn package of care and related supply/equipment needs.
Service delivery	Apply the life course continuum of care to gradually expand access to small and sick newborn care at facilities and referrals from the community through the community-based newborn care approach.	Provide no-cost, quality newborn care services at public health facilities along with introductions of various other schemes (like JSY, JSSK, PMSMA, SUMAN initiative) to reduce out-of-pocket expenditures and wage loss for parents.^67^^,^^108^	Introduce services for small and sick newborn care at district hospitals in a stepwise manner—focusing first on KMC, then CPAP, and finally integrated care.^121^^,^^127^^,^^131^^,^^136^^,^^145^^,^^157^	Test a model of neonatal care in limited sites and then scaling successful results at national level.^164^^,^^182^^,^^183^
Family-centered care	Communicate and counsel both mother and husband for informed decision.	Introduce a national policy to integrate family-centered care in all SNCUs.^104^^,^^105^	Care for the mother-baby dyad as a unit.^155^^,^^161^^,^^162^	Create an enabling environment for family members to become involved in provision of newborn care.

Abbreviations: CPAP, continuous positive airway pressure; FBNC, facility-based newborn care; HMIS, health management information system; JSSK, Janani Shishu Suraksha Karyakaram; JSY, Janani Suraksha Yojana; KMC, kangaroo mother care; NBCC, newborn care corner; NBSU, newborn stabilization unit; NICU, neonatal intensive care unit; PMSMA, Pradhan Mantri Surakshit Matritva Abhiyan; SNCU, special newborn care unit; SUMAN, Surakshit Matritva Aashwasan.

Across all 4 countries, building the capacity of a specialized workforce, measuring neonatal-specific indicators, and adapting existing infrastructure were used as strategies to support small and sick newborn service delivery.

Workforce training was a key strategic priority in Malawi, where the longstanding practice of nursing rotation was addressed by (1) identifying that no policy for nursing rotation was in place, (2) creating staffing rotations and transfer schedules that allowed for continuity of small and sick newborn care by trained specialized staff, (3) avoiding rotation of health personnel who had been trained in neonatal care from neonatal facilities to non-neonatal facilities, and (4) building a case with district health management teams to freeze rotations of personnel experienced and trained in care for small and sick newborns and to designate staff for neonatal units. In Ethiopia, a quality improvement approach developed by the Ethiopian Pediatric Society, in collaboration with the Institute for Healthcare Improvement, that pairs education with external mentorship to support facility-based quality improvement was tested.^222^ In Rwanda, the Human Resources for Health program focused on building capacity among pediatricians and neonatal nurses. In India, training consisted of a 4-day training for medical officers and staff nurses posted in district hospital special newborn care units (SNCUs) followed by a 2-week observership in an SNCU collaborative center or a medical college hospital with a level-3 neonatology unit.

Information systems were strengthened to capture and use neonatal data to improve quality of care. For example, Malawi introduced an online system to track maternal and neonatal outcomes (MATSURV). Also, the Ethiopian Neonatal Network, in collaboration with the Vermont Oxford Network, was created to use data captured on REDCap (Research Electronic Data Capture) to improve the quality of neonatal care through supportive supervision and mentorship and periodic review meetings at national and subnational levels.^223^

Efforts to upgrade infrastructure appeared to follow the standardized guidelines developed in India for SNCUs (i.e., 12 beds per 3,000 deliveries and 4 beds for additional 1,000 deliveries, with approximately 50 square feet per bed for patient care areas and 50 square feet for ancillary care). For example, Ethiopia has adapted its neonatal intensive care unit (NICU) layout from the floor plan currently used in India.

Strategies to finance inpatient small and sick newborn care were distinct. The Ethiopian strategy was to subsidize the cost of care in public health facilities and implement community-based health insurance schemes.^224^ In India, financial resources were provided to states in a 60:40 (national/state) sharing ratio using annual program implementation plans to develop budgets for facility-based newborn care. In Malawi, the focus was on advocacy for subnational budget allocation. In Rwanda, care was fully funded through universal health coverage.

Ensuring availability of essential medical supplies and equipment by improving supply chain logistics was a priority. Malawi introduced security measures, such as an electronic tracker system for equipment used in the small and sick newborn care units. In addition, priority was given to negotiating service contracts for regular equipment maintenance and extended warranties with equipment manufacturers when possible. India encouraged collaboration of neonatologists, engineers, and entrepreneurs to produce and supply high-quality neonatal equipment of several high-volume categories at affordable cost.^225^ Appropriate technology was also developed collaboratively in Malawi (e.g., Pumani bubble continuous positive airway pressure [bCPAP]) and Rwanda (e.g., Dreamwarmer non-electric infant warmer).

Strategies to expand service delivery to the district level differed substantially by country. In Ethiopia, the life course continuum of care was applied to gradually expand access to small and sick newborn care at facilities, along with bolstering referrals through community-based newborn care. The Indian strategy to expand care included provision of no-cost, quality newborn care services at public health facilities, along with other schemes to reduce out-of-pocket expenditures and wage loss for parents. SNCUs were operationalized at district levels, and care was extended to the subdistrict levels by establishing newborn stabilization units at the first referral units and community health centers. Malawi introduced services for small and sick newborn care at district hospitals in a stepwise manner—focusing first on kangaroo mother care (KMC), then CPAP, and finally, integrated care. Conversely, in Rwanda, a model of small and sick newborn care was developed and tested at the district hospital level in the country’s more remote, rural eastern region and then transferred back to the tertiary level in the capital and nationally.

### Innovations Used to Support the Establishment of Small and Sick Newborn Care

To ensure feasible adaptation to local health systems, country stakeholders innovated a myriad of solutions to support the establishment of small and sick newborn care (Table 2).^226^^–^^228^

**TABLE 2.** Innovations Used to Support the Establishment of Small and Sick Newborn Care, by Health System Building Block and Country

Health System Building Block	Ethiopia	India	Malawi	Rwanda
Leadership and governance	Using the National Newborn and Child Survival technical working group and stakeholder coordination meetings to harmonize plans, avoid duplication of efforts and mobilize resources to strengthen newborn and child survival interventions.	Identify high-priority districts, high-focused districts, and aspirational districts by the Ministry of Health and Family Welfare for focused interventions based on maternal and newborn health indicators to address equity gaps.	Newborn focal person to create a platform for discussing and planning for small and sick newborn care at both the national (Ministry of Health) and district (facility and ward) levels.	High-level government leads and owns activities to ensure programmatic sustainability.
Human resources	Task-shift small and sick newborn inpatient NICU care from doctors to trained nurses.^16^^,^^226^	National and regional collaborative centers that provide leadership and technical guidance to ensure uniform, high-quality introduction and implementation of facility-based newborn care.	Implement the COIN curriculum in preservice training for all health providers (including clinical officers) that do not receive directed training on small and sick newborn care.	Develop new cadres and staffing models, including partnership with Rwanda Pediatric Association for a continuous mentorship model.
Information systems	Integrate perinatal and maternal death surveillance and response into the national public health emergency management system.^54^	The SNCU online portal, a real-time data monitoring system, records vital information on the performance of SNCUs in the country as well as the long-term outcomes of discharged neonates; these data are used for guiding policy and initiating action for improving perinatal care.	A robust information system using the NEST360 platform and facility quality improvement dashboard to summarize outcomes of clinical care, enabling stakeholders to improve the quality of service.	Use electronic data management systems like electronic medical records, RapidSMS-MCH,^176^^,^^227^ and RapidPro^228^ to improve data management system (in general) and use of SMS-based system to notify mothers and newborns with danger signs in the community who need urgent care.
Infrastructure	Establish a newborn corner in all labor and delivery wards and a NICU at hospitals for comprehensive small and sick newborn care.^13^^,^^36^	Situate comprehensive lactation management centers close to SNCUs to ensure access to mothers’ own milk or donor human milk for admitted infants.	Create a maternity and neonatal unit in a dedicated building that is separate from other clinical services.	Bring the neonatal unit close to the obstetrics unit to avoid transfer delay and newborn hypothermia.
Health system financing	Enhance financial risk protection to access essential health services free of charge through mechanisms such fee waiver and exemption programs.	Direct benefit transfer scheme transfers cash entitlements directly to Aadhaar-seeded bank accounts of all eligible beneficiaries, such as Accredited Social Health Activists and contractual staff to further reduce delays in payments and corruption practices.	Leverage the advocacy skills of civil society and learnings from the family planning sector, which has secured budget line items and higher levels of funding in Malawi.	Establish community-based health insurance so services are accessible to all.
Medical supplies and devices	A web-based medical equipment management information system to support efficient use and proper management.	Train existing cold chain handlers to repair and maintain equipment in the SNCU. This approach was developed in Maharashtra state and then was replicated in other states.	Standardize the specifications of equipment that facilities can purchase or receive as donations to facilitate maintenance; make sure spare parts are provided and assess 7% of device cost for servicing.	An innovative neonatal transport and the DreamWarmer are 2 examples of innovation cocreated in Rwanda.
Service delivery	Health extension workers provide community-based newborn care that includes identifying newborn danger signs, treatment, and referral linkages to health center and hospital levels.	LaQshya certification of health facilities to improve quality of care during delivery and immediate postpartum period, focusing on enhancing satisfaction of beneficiaries, positive birthing experiences, and providing respectful maternity care to all pregnant women attending public health facilities.	Village health clinics that are staffed by health surveillance assistants who are able to identify and refer small and sick newborns.	Use community health workers for early identification of pregnant women, support for antenatal care visits, postnatal and follow-up; creation of center of excellence for NICUs in provinces.
Family-centered care	Family counseling tool in local language for health extension workers/health workers to counsel caregivers through the continuum of care at community and health facility levels. This tool provides an avenue to raise awareness among families about the importance of linkage to inpatient services for small and sick newborns.	Mother-neonatal intensive care unit where the mother’s bed is placed by the infant’s warmer in the NICU to support zero separation between mother and infant and maternal involvement in taking care of her own baby under guidance of staff. The mother-neonatal intensive care unit is a collaborative effort between the neonatology and obstetrics departments in each facility.	Keep mother and baby together as much as possible when in a facility.	Establishing a policy to encourage the involvement of husbands in labor, delivery, and newborn care. A “companion of choice” is included in the current newborn protocol. Curtains for privacy are provided for each family.

Abbreviations: COIN; Care of the Infant and Newborn; NICU, neonatal intensive care unit; SMS, short message service; SNCU, special newborn care unit.

Overall, incorporating a specific focal person for newborn care and a supporting institutional structure, such as a technical working group, were used for coordination. Innovative staffing models recognized the need for a dedicated cadre of trained NICU staff and supported task-shifting care to trained nurses. To support task-shifting, neonatal care training packages were developed, notably the Care of the Infant and Newborn^140^ course implemented in Malawi. In Rwanda, after the genocide, alliances were formed with professional associations (e.g., Rwanda Pediatric Association and the Royal College of Paediatrics and Child Health) to meet human resource needs in facilities, such as trained medical staff that included pediatricians and neonatologists.

Innovative staffing models recognized the need for a dedicated cadre of trained NICU staff and supported task-shifting care to trained nurses.

Across all countries, information systems were upgraded to manage data in an electronic and/or interactive platform, and perinatal and maternal death surveillance and response systems were implemented. Infrastructure innovation ranged from moving the neonatal and obstetrics units closer together to constructing newborn corners and comprehensive lactation management centers. New health financing schemes emerged, such as community-based health insurance and fee waivers that improved access to care and direct benefit transfer schemes that supported the retention of skilled staff. New ways to manage the efficient and proper use of medical supplies and devices were developed, including a web-based medical equipment management system in Ethiopia and training existing cold chain handlers to repair and maintain equipment in the SNCU in India. Provision of services at the community level was enhanced to support the referral of high-risk pregnancies and the management of small and sick newborns once they are discharged from the facility. Family-centered care was incorporated into the existing health system through policy directives that encouraged the involvement of husbands in labor, delivery, and newborn care and that implemented “no separation of mother and baby,” such as in the mother-NICU.^101^

### Distinct Approaches to Roll-Out of Small and Sick Newborn Care Services

We identified 4 distinct approaches to the roll-out of small and sick newborn care services in each country: (1) Ethiopia used a stepwise expansion from a newborn care corner to community-based newborn care to NICU, (2) India used a model newborn care unit as an exemplar for national scale, (3) Malawi used a hub-and-spoke model initiated through KMC and using the introduction of CPAP as a grounding point, and (4) Rwanda introduced small and sick newborn care through a pilot at the district level in a peripheral region as a basis for national roll-out.

In Malawi, a hub-and-spoke model (i.e., piloting at a major urban center and referral hospital and then expanding into select regional locations, eventually reaching across all districts in the country) was used for the roll-out of small and sick newborn care, using the introduction of CPAP as a foundation.

**FIGURE 6:**
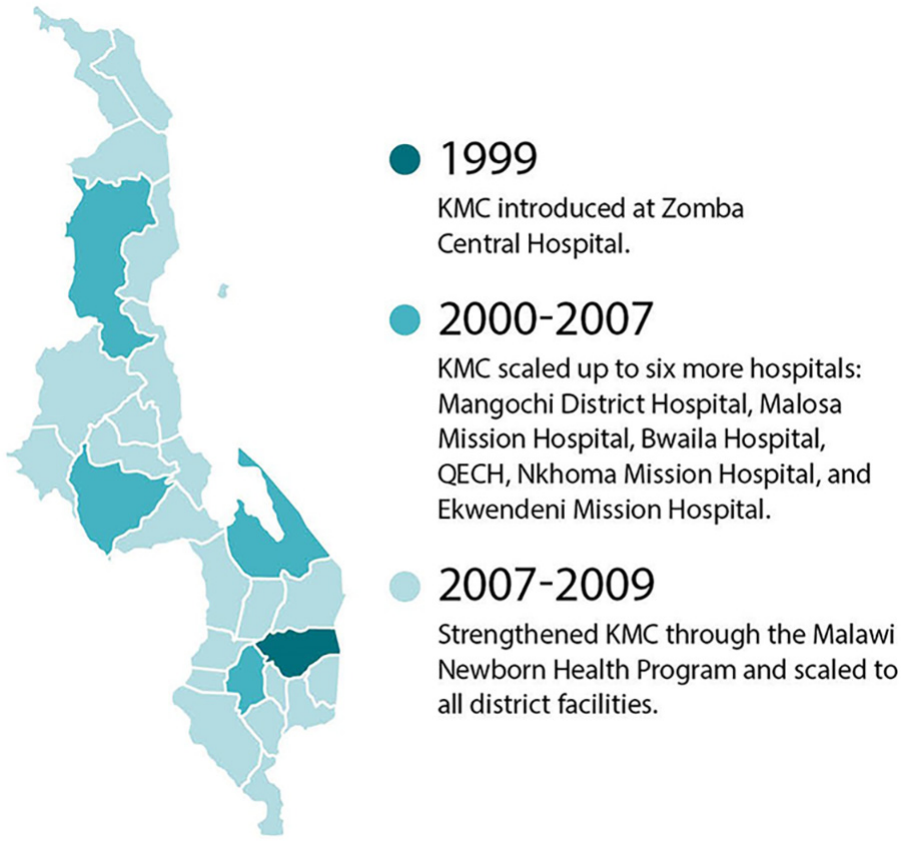
Scaling Up Kangaroo Mother Care in Malawi, 1999–2009 Abbreviations: KMC, kangaroo mother care; QECH, Queen Elizabeth Central Hospital.

**FIGURE 7:**
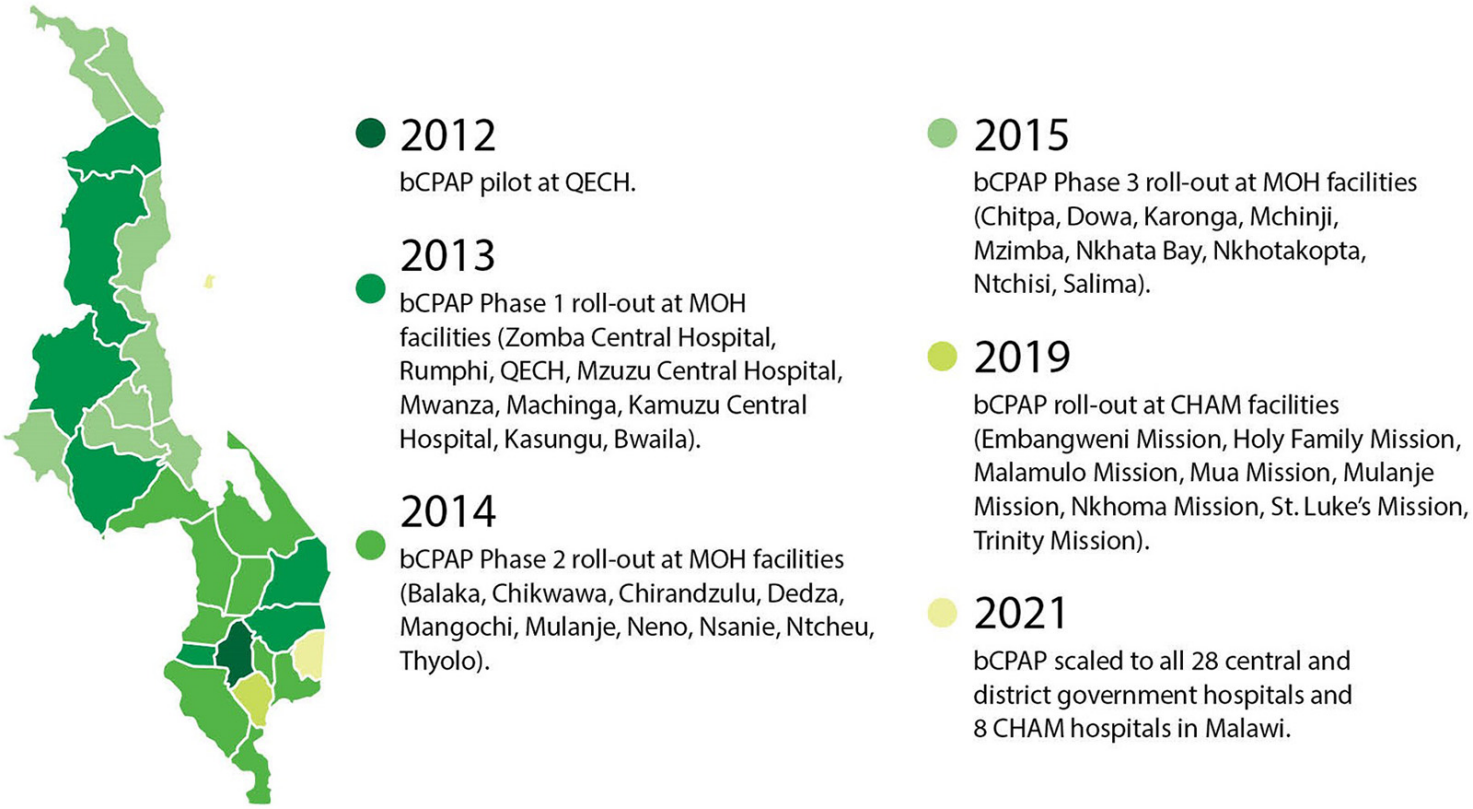
Scaling Up bCPAP in Malawi, 2012–2021 Abbreviations: bCPAP, bubble continuous positive airway pressure; CHAM, Christian Health Association of Malawi; MOH, Ministry of Health; QECH, Queen Elizabeth Central Hospital.

**FIGURE 8:**
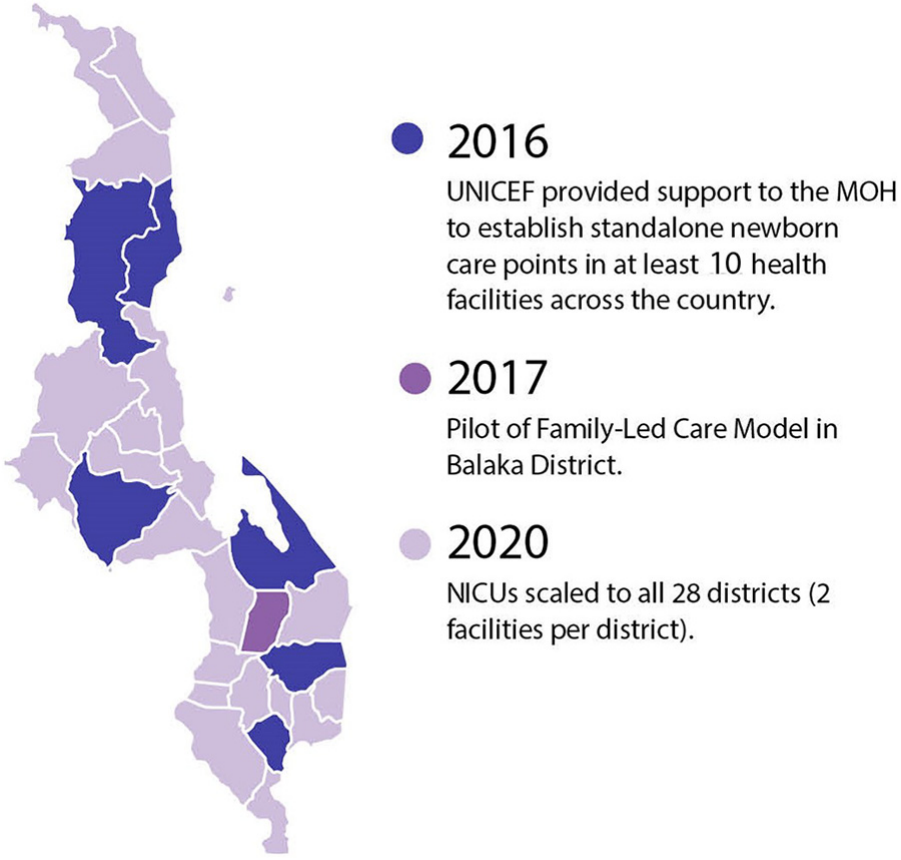
Scaling Up Neonatal Care in Malawi, 2016–2020 Abbreviations: MOH, Ministry of Health; NICU, newborn intensive care unit.

Services for small and sick newborns rolled out over time, beginning around 1999 when KMC was introduced at Zomba Central Hospital, until today. In 2004, KMC was included in the national policy, and in 2005, national guidelines^229^ and a training manual for KMC^230^ were released (Figure 6). Meanwhile, in 2006, Rice University partnered with Queen Elizabeth Central Hospital in Blantyre to help solve the problem of severe respiratory failure in newborns by designing a low-cost bCPAP device. In 2009, just 10 years from the initial introduction, KMC national guidelines were revised,^231^ and the intervention had been scaled to all district hospitals. In 2012, the community-based maternal newborn package of care was introduced to further support KMC. In 2014, an evaluation of Malawi’s progress toward the Millennium Development Goal 4 highlighted a renewed need to focus on neonatal health. The Ministry of Health realized that neonates received care in inappropriate places and that KMC was not being delivered effectively for unstable small and sick newborns. An initial pilot of bCPAP took place at Queen Elizabeth Central Hospital in 2014. In 2015, the ENAP for Malawi was created to ensure that functional newborn units exist in all districts (Figure 7). In 2016, UNICEF provided support to the Ministry of Health to establish standalone newborn care points in at least 10 health facilities. WHO supported 11 districts to develop NICUs, and bCPAP was scaled to all district hospitals. In 2017, a family-led care model—which positioned caregivers and families as active participants in the care of preterm and low birth weight babies in the health facility and at home—was piloted in Balaka District, and, by 2020, district-level inpatient care facilities for small and sick newborns were scaled fully in the country (Figure 8).

**FIGURE 9:**
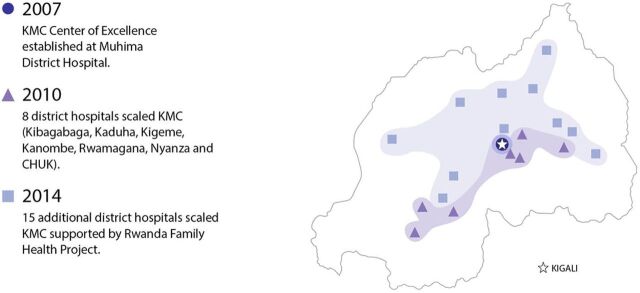
Scaling Up Kangaroo Mother Care in Rwanda, 2007–2014 Abbreviations: CHUK, Centre Hospitalier Universitaire de Kigali; KMC, kangaroo mother care.

**FIGURE 10:**
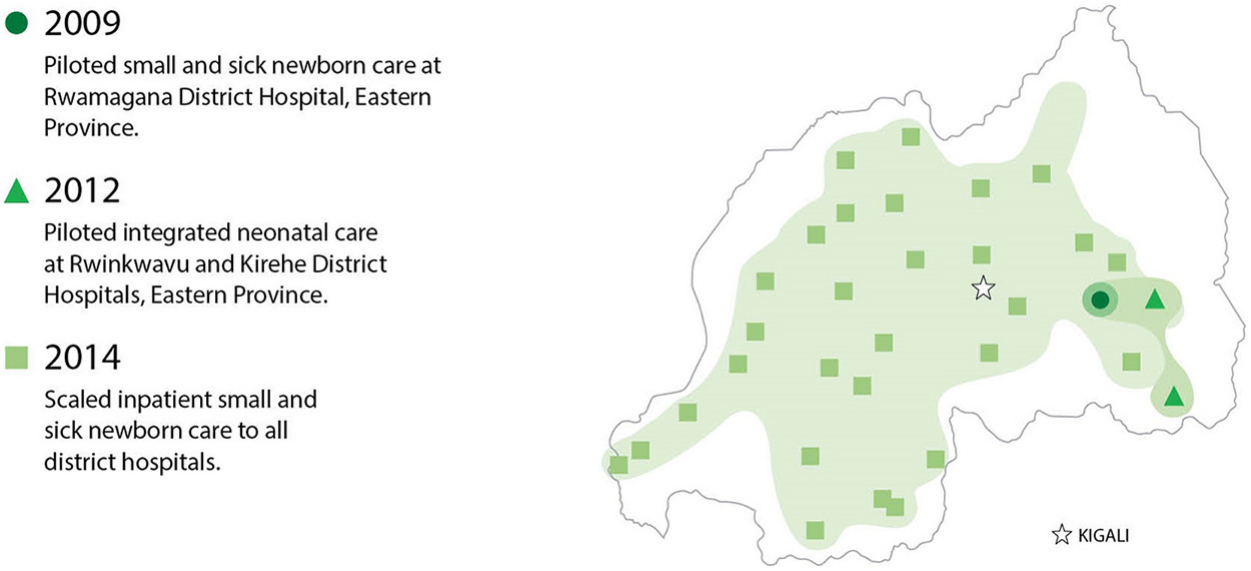
Scaling Up Small and Sick Newborn Care in Rwanda, 2009–2014

In Rwanda, integrated small and sick newborn care was initiated at the district level in the eastern region, far from the capital city. Beginning around 2007, services for small and sick newborns rolled out over time. Services initially focused on KMC, which was introduced as a pilot in 2007 in Muhima District Hospital in Kigali that was established that year as a center of excellence for KMC. From 2007 to 2010, KMC was scaled to 8 district-level facilities. In 2014, KMC was scaled in an additional 15 district hospitals.^195^ In 2009, a more comprehensive approach to small and sick newborn care was piloted in Rwamagana District Hospital. Using the lessons learned from the pilot effort, an integrated neonatal care protocol and standards were developed and implemented in 2 other district hospitals. Based on this experience, the neonatal care protocol and standards were revised and released in 2014 as the National Neonatal Care Protocol, second edition. This effort effectively expanded the reach of small and sick newborn care services into all district hospitals in the country. In 2019, the National Neonatal Care Protocol, third edition, provided updated guidelines for small and sick newborns (Figure 9 and Figure 10).

**FIGURE 11:**
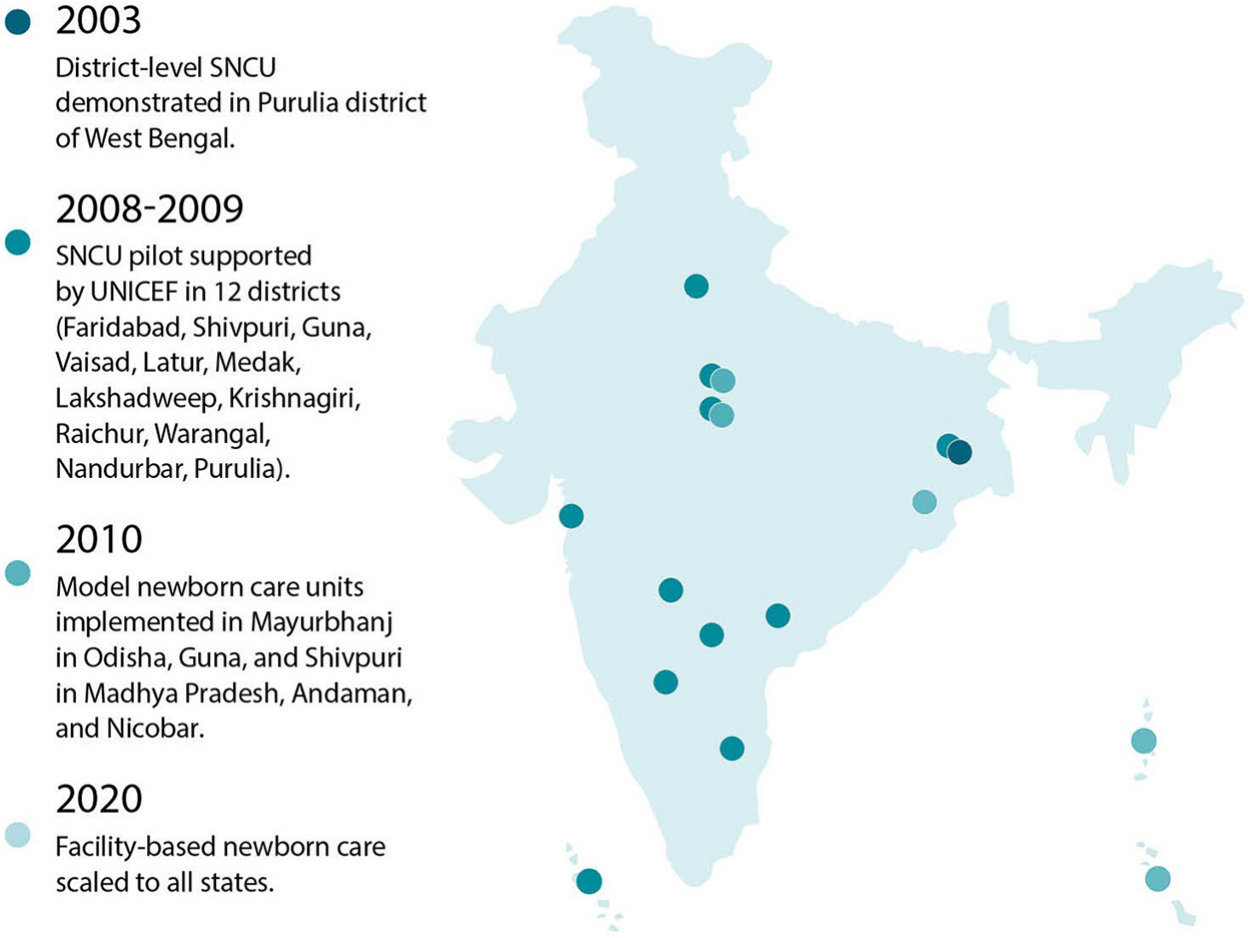
Scaling Up Facility-Based Newborn Care in India, 2003–2020 Abbreviation: SNCU, special newborn care unit. Source: Gupta G. The NICU story of India: pilot to national scale in 10 years. https://youtu.be/WdXHsC-cks4

In India, district-level services for small and sick newborns rolled out over time, beginning in 2003. The feasibility of establishing and operating a district-level SNCU was demonstrated in Purulia district of West Bengal in 2003. The Government of India adapted the Purulia district model for a national model by operationalizing SNCUs at district levels and extending the care of sick newborns to the subdistrict levels by establishing newborn stabilization units at the first referral units and community health centers. In addition, a dedicated space (newborn care corner) was ensured at all delivery points for strengthening essential newborn care and resuscitation. In 2008–2009, UNICEF supported pilot implementation of the SNCU in 12 districts (Faridabad, Shivpuri, Guna, Vaisad, Latur, Medak, Lakshadweep, Krishnagiri, Raichur, Warangal, Nandurbar, Purulia). This experience demonstrated the viability of expanding this small and sick newborn care model in India, and the model was included in the National Rural Health Mission. Next, UNICEF, the Indian Academy of Pediatrics, National Neonatology Forum, and other professional stakeholders worked together to implement model newborn care units in Mayurbhanj in Odisha, Guna, and Shivpuri in Madhya Pradesh, Andaman, and Nicobar. The Government of India created operational guidelines,^81^ which included set up, costing, and steps for establishing newborn care facilities, and developed and launched the facility-based newborn care package and implementation plan in 2011 (Figure 11). By 2020, facility-based newborn care was scaled across the country.

**FIGURE 12:**
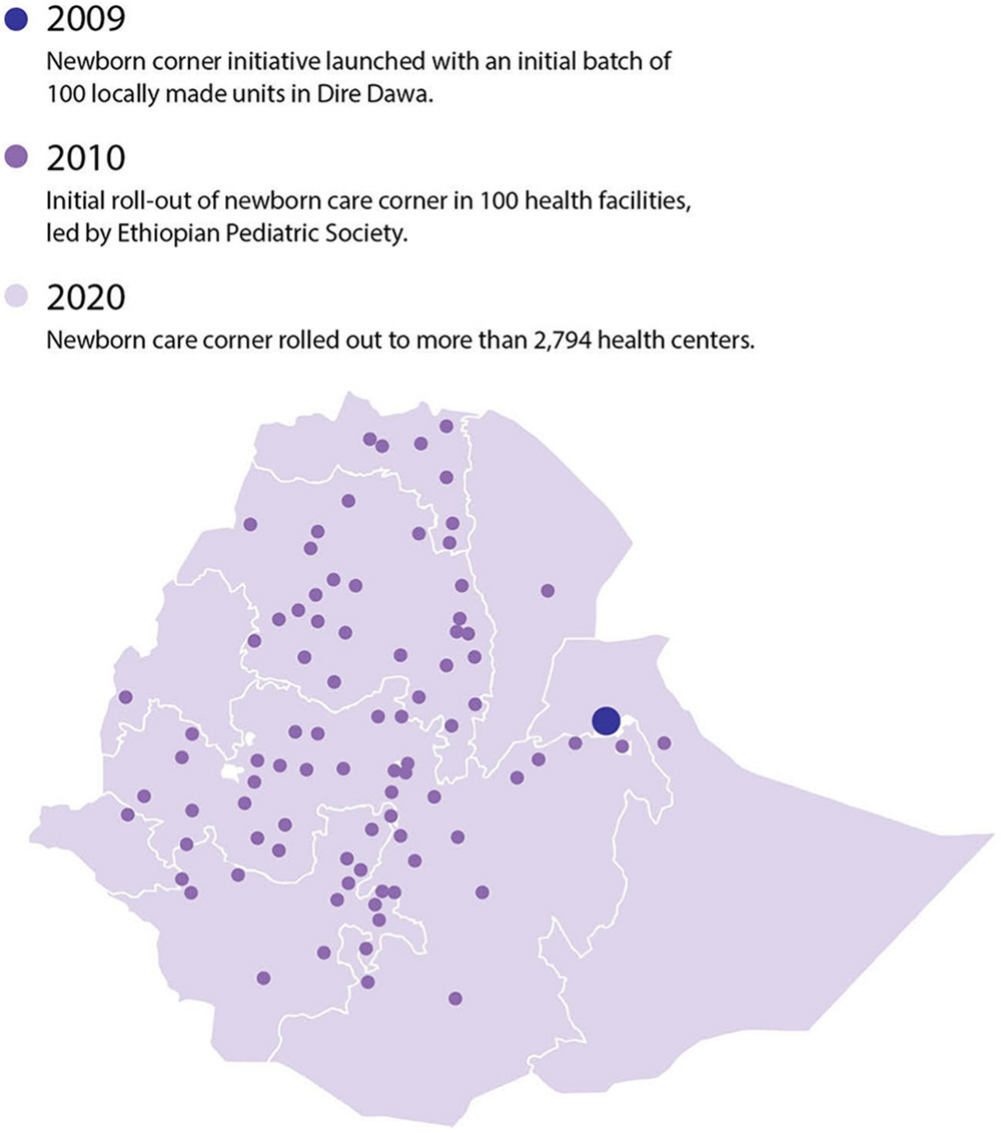
Initial Roll-Out of Newborn Care Corners to 100 Health Facilities in Ethiopia, 2009–2020 Source: Nigussie and Worku.^13^

In Ethiopia, services for small and sick newborns rolled out over time, beginning around 1996 when KMC was introduced at Black Lion Hospital. Over the next decade, services for small and sick newborn care were limited to specialized tertiary facilities where the quality of care was marginal. In 2003, the Ethiopia Federal Ministry of Health (FMOH) began implementing the community health extension program, primarily through health extension workers, to improve access to health services in rural and remote regions. In a corollary effort to expand and strengthen care for small and sick newborns, KMC was included as an annex in the first edition of the Standard Treatment Guidelines for district hospitals.^13^ In 2005, a study on facility-based KMC showed that facility-based KMC was feasible and acceptable to mothers and that survival of preterm low birth weight infants was better than the conventional method of care.^232^ In 2006, Integrated Management of Newborn and Childhood Illnesses launched and included KMC for preterm or low birth weight babies, neonatal resuscitation, infection prevention, and advanced life support in referral hospitals. In 2009, the FMOH, UNICEF, and the Ethiopian Pediatric Society piloted a newborn care corner—which consisted of a hard table, attached overhead heat and light sources, drawer to store essential supplies, self-inflating ambu bag, and face masks in different sizes—that was easy to disassemble and reassemble for cleaning and disinfection in 100 health facilities (Figure 12).^233^ Based on positive results, the newborn care corner was rolled out to the majority of health centers and hospitals beginning in 2010.

**FIGURE 13:**
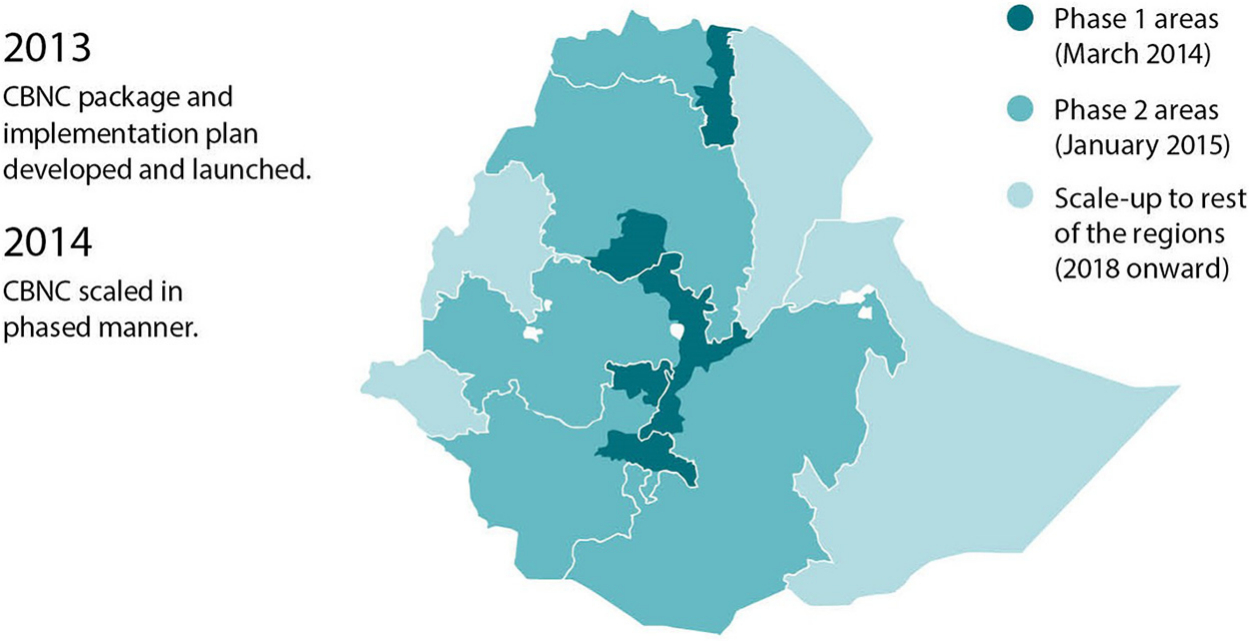
Phased Scale-Up of Community-Based Newborn Care in Ethiopia, 2013–2014 Abbreviation: CBNC, community-based newborn care.

In 2012, an expanded focus on community-based newborn care within the health extension program was phased in (Figure 13). Also in 2012, the FMOH and UNICEF constructed a neonatology unit, the first of its kind in Ethiopia, at the Yekatit 12 Hospital, a public referral hospital in Addis Ababa. The NICU at Yekatit 12 Hospital also became a center of excellence in 2012, and NICU services were expanded to an additional 8 facilities. In 2013, NICU services expanded to another 27 facilities. Then, in 2014, based on this experience, the FMOH developed a training package for NICU services at district hospitals and a NICU implementation guideline in 2016. Also in 2016, the use of advanced technology—2 radiant warmers (General Electric Lullaby warmer), a bCPAP device, 2 high-performance phototherapy devices (General Electric Lullaby), a neonatal monitor (General Electric Carescape V100), resuscitation support laryngoscope, and thermal support to transfer neonates from the labor and delivery ward to the NICU—within NICUs was piloted.^42^ In 2018, the FMOH drafted minimum requirements for NICU services that serve as a reference (e.g., human resource and equipment readiness) for hospital management teams when they establish a NICU and guide procurement of NICU equipment at the national level, as well as capacity-building at all levels. As of 2019, more than 353 hospitals were functional in the country, of which 196 (56%) provided NICU services. Throughout this period, KMC and NICU services have been recognized as high-impact interventions for low birth weight and preterm infants and integrated into policy and guidance documents, most notably the Newborn and Child Survival Strategy 2015–2020, Health Sector Transformation Plan I and II, and National Healthcare Quality Strategy.

We worked with country stakeholders to assess qualitatively the status of implementation of the recently released 2020 WHO Standards for Improving the Quality of Care for Small and Sick Newborn in Health Facilities^9^ (Table 3).

**TABLE 3.** Gap Analysis of Implementation of 2020 WHO Standards for Improving the Quality of Care of Small and Sick Newborns in Health Facilities, by Country

**WHO Standard**	**Rwanda**	**Malawi**	**Ethiopia**	**India**
**1.22** NEW: Small and sick newborns are assessed for surfactant deficiency, and surfactant replacement therapy is administered to preterm newborns within the first 2 hours of birth according to WHO guidelines.		X	X	X
**1.23** NEW: Small and sick newborns at risk of bronchopulmonary dysplasia are assessed, investigated, and managed as per standard guidelines.		X	X	X
**1.25** NEW: Small and sick newborn who cannot tolerate enteral feeding or for whom enteral feeding is contraindicated are provided with parenteral nutrition in correct amounts and composition according to standard guidelines.		X		
**1.27** NEW: All very low birthweight newborns are given vitamin D, calcium phosphorus, and iron supplements, according to WHO guidelines.		X		
**1.32** NEW: Small and sick newborns at risk of necrotizing enterocolitis are assessed and managed according to WHO guidelines.				X
**1.33** NEW: Small and sick newborns at risk of retinopathy of prematurity are appropriately identified, screened, and treated.	X	X		
**1.34** NEW: Small and sick newborns at risk of intraventricular hemorrhage are assessed and managed according to standard guidelines.		X		
**1.40** All small and sick newborns are assessed routinely for pain or symptoms of distress and receive appropriate management according to WHO guidelines.		X	X	
**3.3** For every newborn referred or counter-referred within or between health facilities, there is appropriate information exchange and feedback to relevant health care staff.			X	
**3.5** NEW: Newborn transfer services provide safe, efficient transfer to and from referral neonatal care by experienced, qualified personnel, preferably specialist transport teams, in specialist transport vehicles.		X	X	
**4.4** NEW: Carers of small and sick newborns and staff understand the importance of nurturing interaction with the newborn, recognize and respect the newborn’s behavior and cues, and include them in care decisions.				X
**4.6** NEW: In humanitarian and fragile settings, including outbreak and pandemic situations, special consideration is given to the specific psychosocial and practical needs of small and sick newborns and their carers.	X	X	X	X
**5.4** All newborns are protected from any physical or mental violence, injury, abuse, neglect or any other form of maltreatment.			X	
**5.6** NEW: All newborns who die and all stillbirths have their death registered.			X	

Abbreviation: WHO, World Health Organization.

Across all countries, 24 gap areas were identified, with 15 (62.5%) of those relating to completely new standards for care. Gaps in implementation were observed across all countries, with Ethiopia and Malawi reporting relatively more gaps than Rwanda and India. Similarly, gaps in care for small and sick newborns in fragile or humanitarian settings were noted in all countries. All countries except Rwanda noted facing challenges currently in clinical care, such as administration of surfactant replacement therapy and management of bronchopulmonary dysplasia. All countries except India noted lacking equipment designed specifically for the medical care and developmental and emotional support of small and sick newborns; adequate space for KMC; family-centered care; privacy for mothers to express breast milk; and facilities for hygiene, cooking, and laundry. Countries are working to close these gaps in varying ways. For example, in Malawi, a review of guidelines for retinopathy of prematurity is currently in process in collaboration with ophthalmologists to update the current national standard of care small and sick newborns.

Gaps in implementation were observed across all countries, with Ethiopia and Malawi reporting relatively more gaps than Rwanda and India.

As demonstrated, efforts in each country are uniquely distinct. However, across all 4 countries, stakeholders prioritized a similar set of critical actions to support the establishment of small and sick newborn care (Table 4).^234^^,^^235^

**TABLE 4.** Critical Actions to Support the Establishment of Small and Sick Newborn Care as Prioritized by Stakeholders, by Health System Building Block

Health System Building Block	Actions
Leadership and governance	Establish a national strategy and a collaborative team led by the ministry of health to champion it at national and subnational levels. Provide a supportive structure for the public-private partnership in the scale-up of NICUs in private health facilities.
Human resources	Implement task shifting from doctors to trained neonatal nurses who provide NICU services, together with continuous training, to improve quality of care. Manage the rotation of staff in NICUs by developing a cadre of neonatal nurses who are allocated and dedicated to the neonate unit and establish policies that dissuade managers from rotating key staff and instead support staff retention and motivation. Build capacity via continuous quality improvement efforts that include mentoring and supportive supervision through engagement with professional organizations and academia.
Information systems	Invest in and capacitate staff on data collection and data use and conduct regular neonatal data reviews to identify gaps and propose relevant strategies. Use electronic data dashboards or real-time data monitoring systems to facilitate the use of data for decision-making and program improvement.
Infrastructure	Engage multidisciplinary teams, such as hospital planners, clinicians, public health experts, civil and biomedical engineers, and district planners to develop comprehensive guidance on infrastructure layout to enable family-centered care. Invest adequately to ensure that inpatient units are allocated sufficient space in line with established guidance to support highest quality of care for very sick babies, isolated infectious babies, KMC babies, and the provision of human milk.
Health system financing	Advocate for continuous budget allocations to support provision of quality neonatal care, at all levels, with specific focus on district and facility levels. Consider linking to community health insurance, which can remove barriers to accessing health services, while at the same time transform health-seeking behavior.
Medical supplies and devices	Ensure continuity of care by establishing equipment maintenance and replacement plans at all levels of care and standardizing required neonatal supplies for inclusion into routine supply chain requirements. Foster sustainability by collaborating more between scientists, neonatologists, engineers, and entrepreneurs to guide local innovation, manufacturing, and biomedical support to achieve high-quality equipment at an affordable cost.
Service delivery	Establish mentorship opportunities for peer learning and designate a focal point person specialized in small and sick newborn care at each hospital. Strengthen neonatal care by integrating KMC facilities, standardizing referral and follow-up protocols, and using a continuum of care approach to expand care beyond discharge to the community.
Family-centered care	Gain and maintain the confidence of health care providers regarding the impact of family-centered care is important to implement a successful program. Advocate to include parents through a structured capacity-building program to involve parents in bedside processes of care.

Abbreviations: KMC, kangaroo mother care; NICU, neonatal intensive care unit.

## LESSONS LEARNED

Literature that documents the holistic systems approach to the establishment of inpatient newborn care in LMICs is sparse. Details from the country perspective on what it takes to establish, scale up, and strengthen inpatient newborn care services are critical to guiding future implementation, with learnings that incorporate historical information about key moments and timelines, champions, barriers and enablers, strategies, and key advice to other countries embarking on this journey.

Our in-depth case studies attempt to fill this gap by providing a contextualized narrative of how inpatient newborn care was established and scaled from the perspective of key stakeholders who were key drivers of the implementation of small and sick newborn care in each country. We used the NEST360 Theory of Change for facility-based care, which reflects the WHO health system building blocks to frame the case study to align with learning resources^12^ gathered by global stakeholders around the implementation of care for small and sick newborns.

Efforts to establish and scale inpatient care for small and sick newborns in Ethiopia, India, Malawi, and Rwanda over the last decade have led to remarkable success. Each country devised locally relevant, specific strategies to change their “business as usual” practices and improve care for small and sick newborns. Unifying themes about how this has been addressed at the country level are apparent. These include the importance of leadership and governance at the national level to consolidate and coordinate action related to improvements in quality of care for newborns. In this regard, countries employed national coordination committees and a newborn focal person situated within the ministry of health. This collaborative team, led by the ministry of health, created a national strategy and championed it at national and subnational levels.

Dissemination and utilization of data about newborns, such as the ENAP target-setting effort, raised awareness and focus on the newborn and spurred dialogue and action by country stakeholders. This is a marked shift from 20–25 years ago when data on newborns, especially small and sick newborns in LMICs, were virtually nonexistent. The ENAP was an important catalyst for countries to consolidate their action plans for newborn health, and its impact was particularly evident in India and Malawi, where stakeholders created country-specific ENAPs.^133^^,^^236^ Each country invested in and built skills of staff on data collection and data use and institutionalized regular neonatal data reviews to identify gaps and propose relevant strategies. Electronic data dashboards or real-time data monitoring systems facilitated the use of data for decision-making and program improvement.

Adaptation of the existing infrastructure was crucial to establishing quality care for small and sick newborns. Inpatient units were reorganized or constructed to allocate sufficient space in line with established guidance for very sick babies, isolated infectious babies, KMC babies, and the provision of human milk. Although it was not always available at the country level, comprehensive guidance on infrastructure layout to enable family-centered care is essential. Development of this type of guidance can benefit from the engagement of multidisciplinary teams (i.e., hospital planners, clinicians, public health experts, civil and biomedical engineers, and subnational management).

All countries advocated for continuous budget allocations to support provision of quality neonatal care at all levels, with specific focus on district and facility levels. However, the specifics of how each country conducted budget advocacy differed. Donor dependency was noted as a hurdle in some countries, and donor funding was used in different ways. For example, in Ethiopia, national goals define the use of donor funds. However, in Malawi, changes in personnel/corruption allowed donors to identify their own agenda. Health financing schemes, such as community health insurance, helped to remove barriers to accessing health services and, at the same time, transform health-seeking behavior.

Standardizing required neonatal supplies for inclusion into routine supply chain requirements was employed universally. Successfully implementing family-centered care is most advanced in India, which serves as a model of care for other countries. India is also at the forefront of involving the private sector to care for small and sick newborns in a coordinated manner.

It is challenging to capture all the components required to create a functional intervention at scale. Although documentation around efforts to introduce and scale a new intervention exist, they are not always kept in the same central location and do not always illustrate the entire picture. Those working to advance components of small and sick newborn care do not always have the opportunity to review the entirety of complementary efforts that are advancing progress simultaneously. Keeping documentation in a centralized location and using that information to create event timelines that showcase overall country-level effort could be a best practice for documenting the journey. This type of documentation is important because it (1) celebrates successes at the country level, (2) can serve as an example to other countries, and (3) helps the country to assess the status and identify gaps that still need to be addressed. In general, implementing small and sick newborn care is evolving rapidly at the country level. Not infrequently, content specific to small and sick newborns in policy documents reflects only a partial alignment with WHO standards, which may not depict the larger scale of actual implementation and practice.

The case study approach we used to document the journey to scale helps to illustrate the remarkable progress that is being made to improve the quality of small and sick newborn care at the country level. This reporting approach balances the paucity of gray literature and documentation and literature published in peer-reviewed journals with lived experience from stakeholders who were intimately involved in the process. An additional output based on these lessons includes a virtual multimedia experience where any person can “walk through” an existing unit in Malawi that provides care for small and sick newborns.^237^ This virtual experience offers a realistic glimpse into the newborn care unit and its practices, and health care providers comment on how to successfully establish and scale the care of small and sick newborns via a pop-up video feature.

The case study approach we used to document the journey to scale helps to illustrate the remarkable progress that is being made to improve the quality of small and sick newborn care at the country level.

Building on the numerous lessons^234^ across these 4 case studies, the following next steps to strengthen and transform inpatient newborn care globally should be prioritized.

- Identify cost drivers and determine cost effectiveness of best practices to inform future, sustainable, and scalable implementation.
- Build country-level capacity to document local innovations, approaches, and strategies and to disseminate learnings for informing future investments.
- Transform newborn care experiences through a human-centered approach, exploring documenting and linking both facility-based and family-level experiences to improve systems for providing quality neonatal care.
- Further document the scalability of successful programs for new settings to learn and adopt.

### Limitations

Limitations of the case study approach relate primarily to its subjective nature and heavy reliance on qualitative inquiry methods. The case study approach aimed to conduct an exploratory investigation to gather facts about the scale-up of newborn care. It is conceivable that unconscious bias was introduced through the evaluation of what data were included in the case study. To mitigate this, we standardized data collection methods across all countries, using the NEST360 Theory of Change for facility-based care, which reflects the WHO Health Systems Framework as the organizing principle and triangulating data from multiple sources. We continued to interview key stakeholders until data saturation was apparent and did not bind the upper limit of our sample size in any way. Furthermore, data validation sessions were held in each country to ensure that all relevant data points and the country narrative rang authentic, accurate, and true with all country stakeholders. In line with the case study approach, we collected data from a relatively limited number of participants with highly pertinent experience, which may have provided a skewed view of the journey to scale undertaken in each country. Finally, we did not attempt to report on the quality of care being implemented in each county or collect data on programmatic outcomes, which conforms to our study objective of documenting how small and sick newborn care was established in each country.

We also note the lack of standardized reporting for programmatic case studies. For example, the Equator Network^238^ includes multiple guidelines for reporting clinical case studies; however, none for case studies of this nature. As country case studies are increasingly being used, it will be important to standardize criteria for reporting country experience in a case study format.

## CONCLUSIONS

Around 2010, in Ethiopia, India, Malawi, and Rwanda, services for small and sick newborns were available primarily in tertiary care centers. Over the next decade, constellations of events created momentum to establish inpatient care services at the district level in these countries. In Ethiopia, more than half of the over 353 hospitals in the country provide NICU services. In Malawi, district-level inpatient care facilities for small and sick newborns were scaled fully in the country, with NICUs scaled in all 28 districts. In Rwanda, integrated small and sick newborn care has been established in all 42 district hospitals. In India, the number of SNCUs has increased from 263 to 895 over 10 years, exemplifying the level of scale of these services.

The journeys toward establishing inpatient newborn care in Ethiopia, India, Malawi, and Rwanda over the last decade are similar in terms of trajectory yet unique in implementation. Each country devised locally relevant, specific strategies and innovations that addressed identified gaps in programming and health systems. Four distinct approaches to the roll-out of service delivery were identified. The examples of these countries can inspire other more nascent initiatives. From the global perspective, there has not been a uniform funding approach; more effective coordination may be beneficial. Country-level strategies and innovation related to the establishment of small and sick newborn care require greater documentation and recognition. Documentation should give voice to lived country experience, not all of which is fully captured in existing, peer-reviewed published literature.

## Notes

**Peer Reviewed**

**First published online:** August 7, 2023.
